# Cognition in virtual reality: assessing user acceptability and feasibility of virtual reality cognitive screening for older adults

**DOI:** 10.3389/fpsyt.2025.1570594

**Published:** 2025-07-01

**Authors:** Frank Ho-yin Lai, Benjamin K. Yee, Eileen H. J. Wang, Joe Butler, Andrew Graham, Eddie Yip-kuen Hai, Cath Darling, Stephanie Whittington, Julie-anne Lowe

**Affiliations:** ^1^ Department of Social Work, Education and Community Wellbeing, Faculty of Health and Life Sciences, Northumbria University, Newcastle, United Kingdom; ^2^ Mental Health Research Centre, The Hong Kong Polytechnic University, Hong Kong, Hong Kong SAR, China; ^3^ Department of Rehabilitation Sciences, The Hong Kong Polytechnic University, Hong Kong, Hong Kong SAR, China; ^4^ School of Psychology, University of Sunderland, Sunderland, United Kingdom; ^5^ School of Health & Life Sciences, Teesside University, Middlesbrough, United Kingdom; ^6^ Faculty of Health Sciences and Wellbeing, University of Sunderland, Sunderland, United Kingdom

**Keywords:** spatial-working memory, feasibility and acceptance, technology acceptance model, virtual reality, gamified assessment

## Abstract

**Introduction:**

The global demographic shift towards an older population necessitates innovative methods to assess cognitive abilities, particularly spatial working memory, which is crucial for daily living and early detection of neurocognitive conditions like Alzheimer's disease.

**Methods:**

This qualitative study utilised the Virtual Reality Working Memory Task (VRWMT), a semi-immersive VR activity using keyboard navigation, to assess spatial working memory in older adults. Participants were recruited from community centres and categorised by age and technological familiarity. Focus groups evaluated user perceptions based on the Technology Acceptance Model constructs: Perceived Usefulness, Perceived Ease of Use, Attitude Toward Usage, and Behavioural Intention to Use. The study aimed to assess the acceptability and feasibility of VRWMT across diverse populations, examining its navigational simplicity, emotional engagement, and willingness to endorse VRWMT for routine cognitive assessments.

**Results:**

Findings indicated significant variations in perceived usefulness, ease of use, attitude toward using, and behavioural intention to use across different age groups and socio-demographic characteristics. High-technology-familiarity participants found VRWMT easy to use and engaging, while those with low familiarity struggled with navigation and engagement. Socio-demographic factors such as limited digital literacy and lack of standby support impact technology adoption. Higher technological familiarity leads to better acceptance and feasibility of VRWMT.

**Discussion:**

VRWMT can enhance cognitive health monitoring and therapeutic interventions. The results highlighted that personalised pathways and user-friendly interfaces can improve accessibility and engagement, making VRWMT a valuable tool for cognitive assessments, as part of Occupational Therapy, in diverse populations.

## Introduction

The worldwide demographic transition towards an older population underscores the necessity for creative strategies to assess cognitive abilities in older people (1). Among the cognitive capabilities altered by ageing, spatial working memory, which refers to the ability to hold and manipulate spatial details momentarily in a context-dependent manner, is significant for daily living and may indicate early stages of neurocognitive conditions like Alzheimer’s disease (2). Despite its importance, assessing spatial working memory poses challenges with traditional methods due to its intricate nature and the shortcomings of standard test batteries, which are frequently lengthy, lack adaptability, and do not effectively engage diverse groups (3, 4). Conventional cognitive assessments may not adequately account for cultural and linguistic diversity, leading to the potential underdiagnosis or misdiagnosis of individuals from varied backgrounds (5). Ensuring that effective cognitive assessment tools are accessible to all older adults while considering socio-demographic and socio-economic factors promotes health equity and helps to reduce disparities in cognitive health outcomes (6).

Virtual reality (VR) technology offers a promising solution to these challenges. The VR Working Memory Task (VRWMT), inspired by the Morris water maze, transforms spatial working memory assessments into an engaging, immersive format (7). This laptop-based, semi-immersive VR activity uses keyboard navigation instead of a headset. Wang et al. (8) introduced the VRWMT to older adults (7, 8). Unlike traditional methods, VRWMT allows real-time assessment of spatial working memory through quick, single-trial tasks, providing an interactive and dynamic experience. Recent research highlights its sensitivity to cognitive performance and its correlation with established assessments like the Montreal Cognitive Assessment (MoCA), suggesting its potential as a reliable screening tool (8). By incorporating gamified features, VRWMT reduces cognitive fatigue and monotony while maintaining task intensity, enhancing participant engagement and compliance (9, 10). This innovative approach aims to enhance the precision and accessibility of cognitive assessments for older adults, paving the way for more effective interventions and strategies to support cognitive health (11).

**Figure 1:**
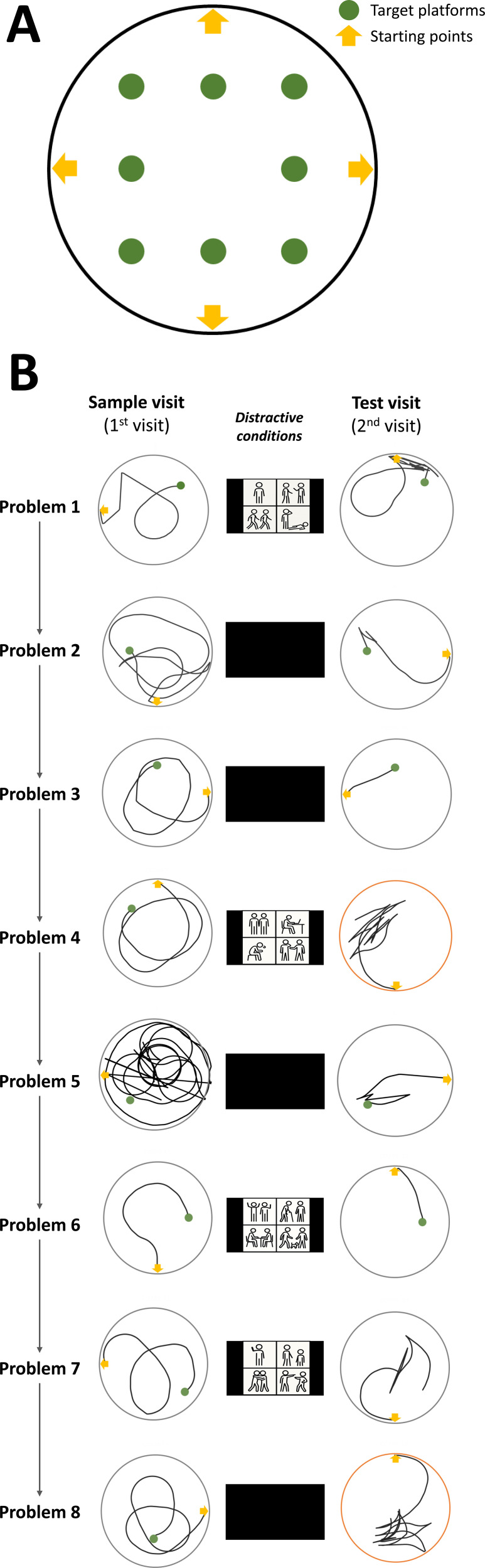
The schematic diagram of the environment and the program of the virtual working memory test (VRWMT). **(A)** Schematic illustration of the circular arena employed in the study. Participants started each visit from one of the four starting points denoted by yellow arrows, while facing the fence. The eight small green circles within the diagram indicate both the locations and dimensions of the potential target platforms. Each target platform was randomly selected and utilized only once throughout the study. **(B)** The VRWMT task contains eight distinct problems. In each problem, participants were required to locate a randomly selected target twice. The two trials to the same target were separated by either a 5-second blank screen or 15-second of four-frame black and white comics (as described in Wang et al. [8]). Notably, the 2nd visit of Problem 4 and 8 served as a 60-second probe test, indicated by orange circles. During these probe tests, the target was removed without informing the participants. The example paths shown here were taken from one young adult from Wang et al. [8].

The VRWMT aligns with the core principles of Occupational Therapy by providing a novel tool for cognitive evaluation and intervention. The integration of VR in Occupational Therapy is increasingly recognised for its ability to create immersive, interactive environments that simulate real-world scenarios (12). VRWMT offers a safe environment for clients to practice and enhance cognitive skills without the risks associated with real-world activities. VRWMT provides instant feedback and performance reports (as depicted in **Figure 1**), enabling occupational therapists to monitor progress and adjust interventions as needed (8). This real-time feedback is crucial for tracking cognitive changes and tailoring therapeutic approaches to meet clients’ evolving needs (13).

Using focus groups, this qualitative study evaluated the application of the VRWMT for assessing spatial working memory. The research evaluated VRWMT’s acceptance and practicality among three age groups: Adults (18–50 years), Younger Elderly (60–69 years), and Older Elderly (70+ years). Based on the Technology Acceptance Model, which was initially introduced by the computer scientists (14) and further validated by health researchers (15), this study aimed to assess the acceptability and feasibility of the use of VRWMT across a diverse population with specific socio-demographic characteristics and technological familiarity. Additionally, the research objectives were to examine the four key constructs of the Technology Acceptance Model: (1) Perceived Usefulness – the tool’s effectiveness in detecting spatial working memory deficits and its potential for early identification of neurocognitive disorders; (2) Perceived Ease of Use – the platform’s navigational simplicity, intuitive design, and user-friendliness for various ages and technological expertise; (3) Attitude Toward Usage – emotional responses such as enjoyment and engagement with the gamified activity; and (4) Behavioural Intention to Use – willingness to endorse VRWMT for routine cognitive assessments in clinical and non-clinical settings. The findings identified barriers and facilitators to adopting the VRWMT to enhance engagement and accessibility for older adults.

Socio-demographic information was collected based on a framework for understanding health disparities (16). A generic socio-demographic classification framework was adapted to categorise individuals into High (H), Middle (M), and Low (L) groups. This classification was determined by employing mixed effects statistical models that combine key socio-demographic variables, including financial status, educational attainment, occupation type, living conditions, access to healthcare, and social support networks. Additionally, technological familiarity was categorised into three tiers: (1) Low: Rarely engages with digital tools and struggles with basic navigation, (2) Moderate: Occasionally uses technology for communication or internet browsing but may face challenges with unfamiliar software, and (3) High: Actively uses technology for various activities such as work, entertainment, or health management. Additionally, the recruited participants were categorised by age demographics: Adults (18–50 years), Younger Elderly (60–69 years), and Older Elderly (70+ years).

In this study, we focus on assessing the user acceptability and feasibility of the tool. It is important to note that this evaluation does not extend to the effectiveness of the intervention itself. While user acceptability and feasibility are crucial for the adoption and implementation of the tool, the ultimate value of the intervention depends on its effectiveness in achieving the desired outcomes. Future studies should aim to rigorously evaluate the effectiveness of the intervention to ensure that it provides tangible benefits to users. Ethics approvals had been granted with reference numbers from the Northumbria University Ethics 44592.

## Methods

### Participants

Participants were recruited via purposeful sampling from December 2021 to March 2024 through poster advertisements at a community centre. Inclusion criteria required participants to have the activity tolerance by participating in the VRWMT for 25 minutes and joining a 90-minute focus group discussion on the next day. Individuals with varying degrees of technological familiarity were included to ensure a diverse range of insights regarding usability. Individuals with motion sickness, motor dysfunction, limited sitting tolerance, or diminished sitting balance (e.g., due to strokes or Parkinson’s disease) were excluded. For safety, the Simulator Sickness Questionnaire (17) assessed virtual reality-induced symptoms before the VRWMT trial.

### Focus group protocol and structure

Each focus group began with a 10-minute casual exchange about participants’ experiences with cognitive games to foster rapport. Researchers FL and CD then presented a brief 10-minute demonstration of the VRWMT, followed by a 25-minute interactive session using the same protocol (8).

After the interactive session, in the day after, an independent facilitator conducted a 90-minute focus group discussion, guided by structured questions to explore key user perceptions: a) Perceived usefulness: Participants discussed the test’s ability to identify cognitive deficits, assist in memory training, and its potential benefits in everyday life or clinical environments, (b) Perceived Ease of Use: Participants responded to three open-ended questions about the interface’s user-friendliness, challenges encountered, and the intuitiveness of instructions and gameplay, (c) Attitude Toward Using: Researchers explored participants’ thoughts on using the test, whether they found it engaging or frustrating, and if they would recommend it to others in their age group, (d) Behavioural Intention to Use: The group discussed their likelihood of using the test regularly and under what conditions (e.g., integrated into health check-ups) they would consider using it. Sessions were audio recorded (with participants’ permission) for precise transcription, and observational notes were taken to capture non-verbal signals and group interactions.

### Justification of sample size

The researcher had begun by recruiting an initial sample of 4 participants from each subgroup (Adults, Young Elderly, and Older Elderly), totalling 12 participants. Focus group discussions had been conducted with these initial participants, and the data had been analysed separately for each subgroup to identify emerging themes and the constructs of the Technology Acceptance Model. A saturation monitoring table had been used to track the themes and constructs discussed in each session. Upon evaluating whether new themes and the constructs of the Technology Acceptance Model constructs were still emerging within each subgroup, the researcher proceeded with additional recruitment when necessary. They had recruited additional participants in small batches for each subgroup (e.g., 4 more participants per subgroup), bringing the total to 8 participants per subgroup. Further focus group discussions had been conducted, and the data had been continuously analysed, updating the saturation monitoring table for each subgroup. After analysing the data from 8 participants per subgroup, the researcher had assessed whether thematic saturation had been reached. When new themes or constructs were still emerging, they had recruited the final batch of 4 participants per subgroup, bringing the total to 12 participants per subgroup. Final focus group discussions had been conducted, and the data had confirmed that thematic saturation had been reached within each subgroup, as no new themes or constructs were identified. The researcher had documented the process, including the number of participants recruited at each stage and the criteria used to determine saturation. By following this iterative recruitment process for each subgroup, the researcher had ensured that the sample size of 36 participants (12 per subgroup) was adequate. Thematic saturation within each subgroup confirmed that all relevant themes and the constructs of the Technology Acceptance Model had been captured, providing a robust basis for the analyses.

The research team then conducted a line-by-line analysis with manual coding of the transcriptions, organising feedback according to the Technology Acceptance Model constructs. The thematic analysis followed Braun and Clarke’s (18), identifying patterns within the data. Venkatesh & Bala’s model further supported the exploration of trends and variations in technology acceptance across age groups (19).

### About the VRWMT

In the 25-minute interactive session, the same one-trial VRWMT was employed as described by Wang et al. (8). Briefly, participants were required to navigate a 3-dimensional circular arena from a randomly selected starting position to locate a target. By utilizing the arrow keys, participants could move forward, backward, and turn left and right within the arena. The location of the potential target platforms and the starting points are shown in **Figure 1A**. During the 1^st^ trial, participants kept searching the arena until the hidden target was located or reached a maximum of 2 minutes. Upon reaching the platform, participants were required to memorize its location and return to the same target as soon as possible in the subsequent trial (second trial). The navigation is exclusively guided by the eight distant landmarks outside the arena. If the target remained undiscovered after 2 minutes, a flag would appear indicating the target’s location and assist the participant in reaching it. Following the second trial, participants sought out a new target, akin to the process in the 1st trial. Each pair of trials directed toward the same target was defined as a single problem, and each participant completed eight such problems, corresponding to the eight distinct targets in a random order (**Figure 1B**). The second trial of the fourth and eighth platforms was the probe test, in which the platform was removed without notifying, and participants were required to search for it over a 60-second interval. Consistent with Wang et al. (8), the first and the second trial were separated either by a 5-second blank condition or a 15-second distractive condition (**Figure 1B**). This manipulation aimed to vary the working memory load experienced by the participants during the task.

## Results

**Figure 2:**
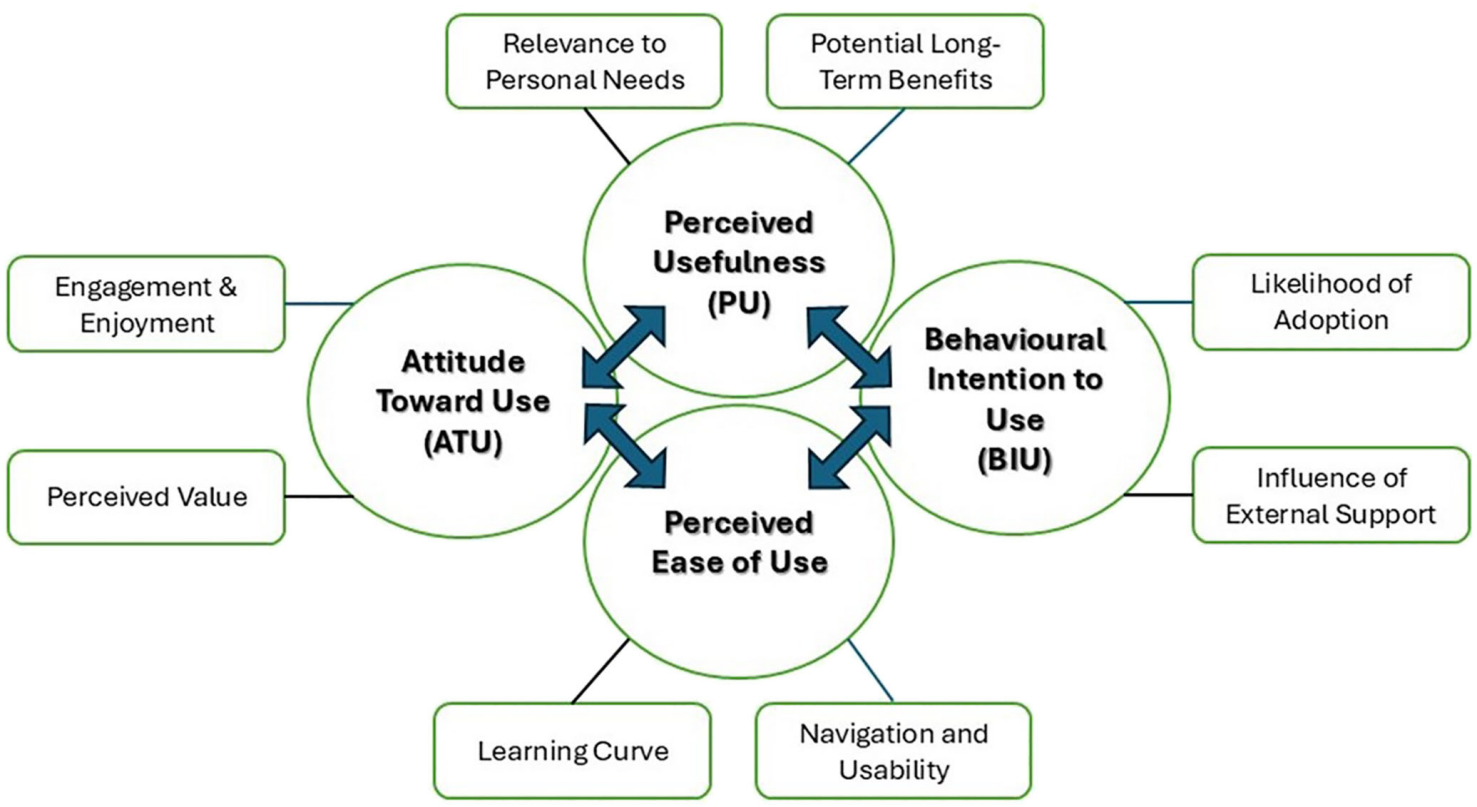
The thematic map created by Braun and Clarke in (18) illustrates four primary themes.

With the recruitment of 36 participants, including 12 adults (18–50 years) with 6 men and 6 women, 12 younger elderly (60–69 years), consisting of 7 men and 5 women, and 12 older elderly (aged 70+), with the grouping of 6 men and 6 women. This section reports the findings from the focus group on the acceptability and feasibility of the use of VRWMT across diverse populations with specific socio-demographic characteristics and technological familiarity. Additionally, to report specific findings of the perceived usefulness, perceived ease of use, attitude toward using, and behavioural intention to use. The narrative analyses of adult, younger elderly and older elderly participants were depicted in **Tables 1**–**3** respectively. Additionally, a thematic map was illustrated in **Figure 2**.

**Table 1:** Narrative analyses of adult (n=12) - (18–50 years).

Code of participants	TF	ED	SD	Perceived Usefulness	Perceived Ease of Use	Attitude toward Using	Behavioural Intention to Use
YA1	H	H	H	“Great tool for tracking memory.” “This app helps me stay sharp and proactive about my mental health.”	“Very easy to use, interface is intuitive.” “It requires no learning”	“Love the approach!” “I enjoy the gamified elements and will recommend it.”	“Would use weekly for tracking.”
YA2	M	M	M	“Could help prevent cognitive decline.” “It’s useful but could be more interactive for better engagement.”	“Took some time to understand instructions.” “Some features felt less intuitive initially, but easy to learn.”	“Neutral, not particularly exciting.” “Not exciting enough to hold my interest.”	“It’s fun and functional.” “Might use occasionally, needs reminders.” “I’d use it occasionally when I feel the need to track my memory.”
YA3	M	L	L	“Not sure how this applies to me.” “I’m not sure how useful it is without more personalized options.”	“Found navigation confusing at times.” “It’s simple, but I still needed help to understand.”	“Not very engaging, feels complicated.”	“Unlikely to use without strong guidance.”
YA4	H	H	M	“Useful for self-monitoring.” “I can see the potential for real cognitive benefits here.”	“Interface is seamless, love the visuals.” “It’s straightforward and well-designed for ease of use.”	“Very engaging and interactive.” “The app aligns well with my self-improvement goals.”	“Would often use to monitor cognitive health.” “I’ll use it regularly to monitor progress over time.”
YA5	M	M	M	“I see its potential for tracking health.” “It’s helpful and offers a good way to keep track of brain health.”	“Not difficult but took a few attempts to adjust.” “The interface was easy to use, even for someone not very techy.”	“Liked the features, but not fully engaging.” “I’d use it if I had reminders to stay consistent.”	“Would try but might forget about it later.”
YA6	M	L	L	“I don’t see its relevant to me.”	“Found some parts confusing.”	“Didn’t find it engaging or fun.”	“Not likely to use.”
YA7	H	H	H	“Perfect for monitoring subtle changes.” “The gamification makes it both engaging and practical for me.”	“Very intuitive, enjoyed using it.” “Very smooth navigation and clear instructions.”	“Impressed by the thoughtful design!” “I love using it—it feels rewarding and beneficial.”	“Adding it to my health tools.” “I’ll integrate it into my activity routine.”
YA8	M	M	L	“Seems useful but unsure about long-term impact.”	“Not hard but required some guidance.”	“Neutral, liked some features but not engaging.”	“Might use occasionally if prompted.”
YA9	H	H	H	“Very valuable for tracking and feedback.”	“Simple to navigate, easy to understand.”	“Really like the gamified testing approach.”	“Would integrate into routine assessments.”
YA10	L	M	L	“Not sure if it’s relevant to me yet.”	“Felt somewhat tricky at first.”	“Okay but didn’t find it engaging.”	“May not return to it unless it’s for a study.”
YA11	H	H	H	“I see its value but not convinced yet.”	“Not very difficult but took effort to adjust.”	“Liked the potential but not very engaging.”	“Might use if recommended.”
YA12	M	L	L	“It’s okay but it is not relevant to me.”	“Found some parts confusing.”	“Didn’t find it engaging or fun.”	“Not likely to use.”

TF, Tech Familiarity; H, High; M Moderate; L, Low.

ED, Education Levels; High (postgrad), Moderate (undergrad), Low (high school).

SD, Socio-demographic; H, High; M, Middle; L, Low.

**Table 2:** Narrative analyses of younger elderly (n=12) – (60–69 years).

Code of participants	TF	ED	SD	Perceived Usefulness	Perceived Ease of Use	Attitude toward Using	Behavioural Intention to Use
YE1	L	M	L	“Seems a bit too complex for me.” “It seems helpful, but I’m not sure how to make it part of my routine.”	“Struggled with parts of the navigation.” “Some parts were confusing, and I needed assistance.”	“Okay, but not enjoyable.”	“I’d only use it if someone helped and accompanied me with it.”
YE2	M	H	M	“This can be helpful. It’s a great idea to monitor cognitive health as I age” “It’s a great idea to monitor cognitive health as I age.”	“Simple to navigate, clear visuals.” “Everything worked well; I had no trouble using it.”	“Fairly engaging.” “I’d encourage others in my age group to try it.”	“Might use it occasionally.”
YE3	L	M	L	“I’m not sure if it applies to me. I don’t see much benefit for someone like me”	“Difficult to figure out on my own.” “I didn’t know where to start.”	“Neutral, didn’t find it interesting.” “It’s not very appealing to me; I prefer simpler methods.”	“Wouldn’t use this without assistance.”
YE4	M	H	M	“Good for identifying early cognitive decline.”	“Easy enough to follow.” “Some tasks were a bit tricky, but overall, it’s manageable.”	“Appreciated its purpose.” “It’s enjoyable, and I can see the benefits.”	“Would use it monthly if prompted.” “I’d recommend it to others”
YE5	L	M	L	“Not very useful for my needs.”	“Felt difficult to use.”	“Didn’t enjoy the experience.”	“Wouldn’t return to it.”
YE6	M	H	M	“Could be good for early memory issues.”	“Straightforward after first trial.”	“Liked the feedback system.”	“Would try it monthly.”
YE7	L	M	L	“Unclear how much benefit I’d get.”	“A bit complicated, needed help to start.”	“Not engaging but functional.”	“Wouldn’t use unless heavily supported.”
YE8	M	H	M	“Useful for early interventions.”	“Easy enough to follow after a bit of practice.”	“Found it fairly engaging overall.”	“Would use it as recommended by doctors”
YE9	L	M	L	“Doesn’t seem very helpful for me.”	“Found it confusing to navigate.”	“It’s functional but too clinical.”	“May not return to it unless strongly encouraged.”
YE10	M	H	M	“Could help me track changes over time.”	“Clear instructions, easy to follow.”	“Enjoyed using it.”	“Would use it monthly for tracking.”
YE11	L	M	L	“Feels unnecessary for my needs.” “I like the idea, but I’m not sure how accurate it is.”	“Confusing to use at first.” “Lacked detailed written instructions.”	“Neutral, not very engaging.”	“Unlikely to use unless instructed.”
YE12	M	H	M	“Useful for detecting problems early.”	“Straightforward after initial guidance.”	“Enjoyed learning through the tool.”	“Would try if recommended by a health professional.”

TF, Tech Familiarity; H, High; M, Moderate; L, Low.

ED, Education Levels; High (postgrad), Moderate (undergrad), Low (high school).

SD, Socio-demographic; H, High; M, Middle; L, Low.

**Table 3:** Narrative analyses of older elderly (n=12) - 70+ years.

Code of participants	TF	ED	SD	Perceived Usefulness	Perceived Ease of Use	Attitude toward Using	Behavioural Intention to Use
OE1	L	L	L	“Could help, but I’m not tech-savvy.” “Doesn’t fully understand its usefulness”	“Needed assistance to complete tasks.” “I struggled with the interface.” “If there were a video tutorial or a guide, it would be much easier to learn”	“I like the health benefits idea.”	“Would only use if my children helped me.” “I would use it if it helps me keep track of my memory”
OE2	L	M	L	“I see how it helps, but it feels too advanced.” “If this can help me stay sharp and independent, I’d be interested”	“Struggled with the setup and navigation.” “Can the game be simplified?” “I couldn’t figure out how to move around in the virtual environment”	“Not very engaging, feels cumbersome.”	“Probably wouldn’t use it.” “Unlikely to use it on my own.” “I’d need someone to show me how to use it properly”
OE3	L	L	L	“I don’t think this is for someone like me.” “I’m not sure how effective this is for someone my age.”	“Interface felt too overwhelming.” “The app feels too modern for me to handle alone.”	“Didn’t enjoy the experience much.”	“Wouldn’t use this again.” “I’d only try it if someone taught me how to use it.”
OE4	L	M	L	“It might help, but it feels too advanced for me.” “I’m not sure if this will help me in the long run.”	“Found navigation very tricky.” “I needed a bit of help to get started, but it’s manageable.” “If the buttons were bigger and the instructions were clearer, I might be able to use it”	“Neutral, didn’t enjoy it much.”	“Unlikely to use this again.” “I’d use it with a bit more guidance”
OE5	L	L	L	“Not sure how this applies to my daily life.”	“Complicated and confusing to use.”	“Neutral, felt like too much effort.”	“Wouldn’t consider using it.”
OE6	L	M	L	“Seems like it could be helpful, but I struggled.”	“Needed help to understand the instructions.” “It took me a long time to understand how to navigate the system”	“Okay, but not very enjoyable.”	“Would only use if someone assisted me.”
OE7	L	L	L	“Unclear how this could help me.”	“Found it too overwhelming.”	“Didn’t like the experience.”	“Not inclined to return to it.”
OE8	L	M	L	“Might be helpful, but I need more guidance.”	“Somewhat confusing without help.”	“Neutral, didn’t find it engaging.”	“Unlikely to use unless strongly recommended.”
OE9	L	L	L	“Not relevant to my needs.”	“Complicated to figure out.”	“Didn’t enjoy it much.”	“Wouldn’t use it again.”
OE10	L	M	L	“Could be helpful, but I struggled with parts.”	“Needed help navigating the tool.”	“Okay but not engaging.”	“Might try if strongly encouraged.”
OE11	L	M	L	“I see the value but struggled with using it.” “It’s nice, but I think it’s for younger people.”	“Needed help with every step.” “It felt complicated, and I didn’t enjoy using it.”	“Didn’t enjoy it much.”	“Would need a lot of assistance to use.”
OE12	L	L	M	“Not something I’d use.”	“Confusing interface.”	“Neutral, didn’t engage with it.”	“Not likely to use it.”

TF, Tech Familiarity; H, High; M, Moderate; L, Low.

ED, Education Levels; High (postgrad), Moderate (undergrad), Low (high school).

SD, Socio-demographic; H High; M, Middle; L, Low.

### Perceived usefulness

The perceived usefulness of the VRWMT tool varied significantly across different age groups and socio-demographic characteristics. Among adults aged 18–50 years, those with high socio-demographic status and high technology familiarity found the tool very useful for tracking cognitive health. For instance, YA1 stated, “Great tool for tracking memory,” and YA7 added, “Perfect for monitoring subtle changes.” Participants with moderate socio-demographic status and technological familiarity saw potential but desired more interactivity, as YA2 mentioned, “Could help prevent cognitive decline,” and YA5 noted, “I see its potential for tracking health.” However, those with low socio-demographic status and technological familiarity were unsure of its relevance, with YA3 stating, “Not sure how this applies to me”.

Among younger elderly participants aged 60–69 years, those with high socio-demographic status and moderate technological familiarity found the tool helpful for monitoring cognitive health. YE2 stated, “This can be helpful. It’s a great idea to monitor cognitive health as I age,” and YE10 added, “Could help me track changes over time.” Participants with moderate socio-demographic status and technological familiarity appreciated its purpose, as YE4 mentioned, “Good for identifying early cognitive decline,” and YE6 noted, “Could be good for early memory issues.” However, those with low socio-demographic status and technological familiarity did not see much benefit, with YE3 stating, “I’m not sure if it applies to me”.

For older elderly participants aged 70+ years, those with low socio-demographic status and low technological familiarity generally found the tool not very useful. OE1 mentioned, “Could help, but I’m not tech-savvy,” and OE3 added, “I don’t think this is for someone like me”.

### Perceived ease of use

The perceived ease of use of the VRWMT tool also varied across different age groups and socio-demographic characteristics. Among adults aged 18–50 years, those with high socio-demographic status and high technological familiarity found the tool very easy to use. YA1 stated, “Very easy to use, interface is intuitive,” and YA7 added, “Very intuitive, enjoyed using it.” Participants with moderate socio-demographic status and technological familiarity found some features less intuitive initially but easy to learn, as YA2 mentioned, “Took some time to understand instructions,” and YA5 noted, “Not difficult but took a few attempts to adjust.” However, those with low socio-demographic status and technological familiarity found navigation confusing, with YA3 stating, “Found navigation confusing at times”.

Among younger elderly participants aged 60–69 years, those with high socio-demographic status and moderate tech familiarity found the tool simple to navigate. YE2 stated, “Simple to navigate, clear visuals,” and YE10 added, “Clear instructions, easy to follow.” Participants with moderate socio-demographic status and technological familiarity found it manageable, as YE4 mentioned, “Easy enough to follow,” and YE6 noted, “Straightforward after first trial.” However, those with low socio-demographic status and tech familiarity struggled with navigation, with YE1 stating, “Struggled with parts of the navigation”.

For older elderly participants aged 70+ years, those with low socio-demographic status and low technological familiarity needed assistance to use the tool. OE1 mentioned, “Needed assistance to complete tasks,” and OE4 added, “Found navigation very tricky”.

### Attitude toward using

The attitude toward using the VRWMT tool varied across different age groups and socio-demographic characteristics. Among adults aged 18–50 years, those with high socio-demographic status and high technological familiarity had a positive attitude toward the tool. YA1 stated, “Love the approach!” and YA7 added, “Impressed by the thoughtful design!” Participants with moderate socio-demographic status and technological familiarity were neutral, as YA2 mentioned, “Neutral, not particularly exciting,” and YA5 noted, “Liked the features but not fully engaging.” However, those with low socio-demographic status and tech familiarity had a negative attitude, with YA3 stating, “Not very engaging, feels complicated”.

Among younger elderly participants aged 60–69 years, those with high socio-demographic status and moderate technological familiarity found the tool engaging. YE2 stated, “Fairly engaging,” and YE10 added, “Enjoyed using it.” Participants with moderate socio-demographic status and technological familiarity appreciated its purpose, as YE4 mentioned, “Appreciated its purpose,” and YE6 noted, “Liked the feedback system.” However, those with low socio-demographic status and tech familiarity were neutral, with YE3 stating, “Neutral, didn’t find it interesting”.

For older elderly participants aged 70+ years, those with low socio-demographic status and low technology familiarity had a neutral attitude toward the tool. OE1 mentioned, “Okay, but not enjoyable,” and OE3 added, “Didn’t enjoy the experience much”.

### Behavioural intention to use

The behavioural intention to use the VRWMT tool varied across different age groups and socio-demographic characteristics. Among adults aged 18–50 years, those with high socio-demographic status and high technology familiarity were likely to use the tool regularly. YA1 stated, “Would use weekly for tracking,” and YA7 added, “Adding it to my health tools.” Participants with moderate socio-demographic status and tech familiarity might use it occasionally, as YA2 mentioned, “Might use occasionally, needs reminders,” and YA5 noted, “Would try but might forget about it later.” However, those with low socio-demographic status and tech familiarity were unlikely to use the tool, with YA3 stating, “Unlikely to use without strong guidance”.

Among younger elderly participants aged 60–69 years, those with high socio-demographic status and moderate tech familiarity might use the tool occasionally. YE2 stated, “Might use it occasionally,” and YE10 added, “Would use it monthly for tracking.” Participants with moderate socio-demographic status and tech familiarity would use it if prompted, as YE4 mentioned, “Would use it monthly if prompted,” and YE6 noted, “Would try it monthly.” However, those with low socio-demographic status and tech familiarity were unlikely to use the tool without assistance, with YE1 stating, “I’d only use it if someone helped and accompanied me with it.” For older elderly participants aged 70+ years, those with low socio-demographic status and low technology familiarity were unlikely to use the tool without significant support. OE1 mentioned, “Would only use if my children helped me,” and OE3 added, “Wouldn’t use this again”.

The acceptability and feasibility of VRWMT vary significantly across different socio-demographic characteristics and levels of technological familiarity. Adults with higher socio-demographic status and tech familiarity are more likely to find the app useful and engaging, while older adults and the elderly, particularly those with lower socio-demographic status, may struggle with its complexity and require additional support.

## Discussion

The findings from this study provide valuable insights into the acceptability and feasibility of the VRWMT across different age groups, highlighting the importance of tailoring cognitive assessment tools to meet the diverse needs of users. By examining socio-demographic characteristics and technological proficiency, the study aimed to ensure equitable distribution of cognitive assessment benefits, improving accessibility, acceptability, and effectiveness.

### Situating results in the context of other research

The acceptability and practicality of emerging technologies like VRWMT are significantly influenced by socio-demographic factors. Previous research in occupational therapy and rehabilitation sciences has similarly highlighted the impact of socio-demographic characteristics on technology adoption. For instance, a study found that individuals with limited financial resources face challenges in acquiring necessary hardware and maintaining reliable internet access, creating barriers to widespread adoption (20). This aligns with our findings, where participants expressed concerns about getting access to the community centre to get accessibility of the VRWMT due to financial constraints.

Similarly, our findings echoed with previous findings, which indicated that lower levels of education often result in limited digital literacy, making technology seem complex or intimidating (5). This was evident in our study, where participants with lower educational attainment struggled with the VRWMT’s interface, indicating a need for more user-friendly designs. One participant mentioned, “If there were a video tutorial or a guide, it would be much easier to learn” (OE1). Scholars emphasised the role of previous working experience, suggesting that individuals in physically demanding or low-skill jobs have less exposure to technological advancements (21). Our findings support this, as participants from professional sectors found the VRWMT more accessible and relevant.

Living conditions also impact feasibility, as Hartley (22) highlighted the challenges faced by individuals in unstable or crowded environments, especially in rural areas with poor internet access (22). Access to healthcare is another critical factor; Braveman and Gottlieb (16) found that limited availability reduces opportunities to engage with health technologies, decreasing their perceived importance. Our findings align with this, as participants with limited healthcare access were less likely to engage with the VRWMT (16).

Strong support networks can enhance both the acceptability and the feasibility of the use of new technology (23). Participants in our study who had supportive networks were more likely to adopt and use the VRWMT, as they received assistance with technological setup and encouragement to participate. One participant shared, “I’d only use it if someone helped and accompanied me with it” (YE1).

### Technological familiarity

An individual’s familiarity with technology substantially affects the practicality and accessibility of novel technological applications, particularly among the elderly and those with limited experience with digital tools. A study found that those with greater technological familiarity are more likely to find technology practical, as they can navigate systems easily and troubleshoot minor issues independently (24). This was evident in our study, where participants with higher technological familiarity were more positive about the acceptability and feasibility of the use of the VRWMT, with one participant stating, “It’s a great idea to monitor cognitive health as I age” (YE1).

Conversely, Mariano et al. (25) noted that those with less technological familiarity may encounter difficulties in setup, operation, and comprehension, creating barriers to adoption and sustained use. This issue is especially pronounced among older adults, who often have less experience with advanced technologies and may feel overwhelmed or frustrated by complex interfaces. Our findings support this, as older elderly participants expressed significant challenges in using the VRWMT, indicating a need for simplified interfaces and comprehensive onboarding resources. One older participant noted, “It took me a long time to understand how to navigate the system” (OE6).

### Implications for occupational therapy

The integration of VRWMT into occupational therapy practice presents a significant opportunity to produce substantial empirical evidence regarding the efficacy of VR-facilitated cognitive assessments. A narrative review of immersive virtual reality health games emphasised the potential of VR technologies to support long-term engagement with therapeutic interventions (26), suggesting that subsequent investigative efforts should prioritise longitudinal studies aimed at monitoring the enduring impacts of VRWMT on occupational therapy outcomes, such as cognitive health and functional outcomes. This aligns with our findings, which suggest that VRWMT can significantly enhance cognitive health monitoring.

Studies highlighted the obstacles and enabling factors associated with the adoption of VR technologies in health and social care services (27, 28). Recommendations to frequently identified barriers include technology development to meet user needs more effectively and facilitating VR implementation in clinical settings. Our study suggests that addressing accessibility and requisite training can foster broader implementation of VR-based therapeutic interventions. Research focused on comparative effectiveness can evaluate the relative merits and drawbacks of VRWMT in contrast to conventional cognitive assessment instruments utilised by OT, thereby refining VR methodologies and enhancing their application in clinical settings (29). Additionally, further studies examining user experience and usability can pinpoint particular design elements that either promote or impede user engagement, leading to advancements in both the design and operational functionality of the tool (30).

### Implications of design perspective

To cater to the varying levels of technological familiarity and educational backgrounds among older adults, the VRWMT can incorporate personalised pathways to cognitive function assessment based on a concise technology familiarity questionnaire administered during onboarding. Studies suggested that users with limited familiarity and educational attainment can benefit from straightforward instructions, visual step-by-step assistance, and user-friendly interfaces. Researchers have proposed a variety of learning path personalisation methods using different techniques and approaches (31). Conversely, those with greater technological familiarity and educational achievements might value more comprehensive explanations regarding the scientific principles behind the tool and the cognitive advantages it provides. This strategy promotes inclusivity and accessibility, enabling users to interact with the tool at their own pace while sustaining engagement and usability. Utilising user feedback surveys to gather insights is beneficial (32). Additionally, tracking engagement metrics like task completion rates, session lengths, and drop-off points can pinpoint and resolve usability issues (33).

### Limitation

This study has several methodological limitations that should be considered. The sample size of 36 participants is relatively small, which may limit the generalizability of the results. Additionally, the sample may not fully represent the diversity of the broader population, particularly in terms of socio-economic status, cultural background, and geographic location. The reliance on self-reported data introduces potential biases such as social desirability bias and recall bias, as participants may provide responses they believe are expected or may not accurately recall their experiences with the VRWMT.

The use of the Technology Acceptance Model can be enhanced by integrating insights from cognitive psychology. Future studies could employ dual-process theories to distinguish between fast, intuitive decision-making and slower, more deliberate thinking. This approach suggests that the user’s decision to employ technology is influenced both by an instinctive sense of whether the technology appears user-friendly and by thoughtful evaluations of its usefulness. This blend of intuition and analysis could help explain why perceptions of ease of use and usefulness, which are critical components of Technology Acceptance Model, affect individuals’ attitudes and intentions when interacting with VRWMT.

Additionally, reinforcing Technology Acceptance Model with cognitive psychology and health technology acceptance can be furthered by employing methodological triangulation and objective measurements. Besides subjective measures, such as responses from focus groups, future studies could include objective data like task completion times, error rates, or physiological metrics. This approach allows for capturing a more comprehensive picture of how users interact with technology, yielding insights that go beyond mere self-reported attitudes. It also enables validation of the traditional constructs of Technology Acceptance Model by demonstrating how they correlate with real-world performance and cognitive load during technology use.

Addressing these methodological limitations in future research can significantly enhance the acceptability and feasibility of VRWMT. Increasing the sample size and ensuring a diverse participant pool can improve the generalizability of findings and provide a more comprehensive understanding of how different socio-demographic factors influence technology adoption. Incorporating objective measures alongside self-reported data can mitigate biases and provide a more accurate assessment of user experiences. By addressing these limitations, future research can improve the acceptability and feasibility of VRWMT, making it a more effective and accessible tool for cognitive health monitoring across diverse populations.

## Conclusion

This study explored the acceptability and feasibility of the Virtual Reality Working Memory Task (VRWMT) for assessing spatial working memory in older adults. The findings highlighted significant variations in user perceptions across different age groups and socio-demographic characteristics. Participants with higher technological familiarity and socio-demographic status found VRWMT more useful, easier to navigate, and engaging, while those with lower familiarity struggled with its complexity and required additional support. Socio-demographic factors such as limited digital literacy and lack of standby support were identified as barriers to technology adoption. The study underscored the importance of tailoring cognitive assessment tools to meet the diverse needs of users, promoting health equity and reducing disparities in cognitive health outcomes.

While our findings indicate that the tool is accepted and feasible for use, it is essential to highlight that this study does not evaluate the effectiveness of the intervention. The true value of the tool can only be determined through comprehensive studies that assess its impact on the intended outcomes. Future research should address the methodological limitations, including small sample size and short-term interactions, to further validate the long-term effectiveness and generalizability of VRWMT.

## Data Availability

The data that support the findings of this study are available from the corresponding author upon reasonable request.
